# Emerging technologies for cardiac tissue engineering and artificial hearts

**DOI:** 10.1002/SMMD.20220040

**Published:** 2023-02-16

**Authors:** Lingyu Sun, Yu Wang, Dongyu Xu, Yuanjin Zhao

**Affiliations:** ^1^ Department of Rheumatology and Immunology Institute of Translational Medicine Nanjing Drum Tower Hospital School of Biological Science and Medical Engineering Southeast University Nanjing China

**Keywords:** cardiomyocyte, regenerative medicine, scaffold, stem cell, tissue engineering

## Abstract

Heart diseases, especially cardiovascular diseases, have brought heavy burden on society for their high morbidity and mortality. In clinical, heart transplantation is recognized as an effective strategy to rescue the lives of patients, while it may suffer from lack of donors and possible immune responses. In view of this, tremendous efforts have been devoted to developing alternative strategies to recover the function and promote the regeneration of cardiac tissues. As an emerging field blending cell biology and material science, tissue engineering technique allows the construction of biomimetic living complexes as organ substitutes for heart repair. In this review, we will present the recent progress in cardiac tissue engineering and artificial hearts. After introducing the critical elements in cardiac tissue engineering, we will present advanced fabrication methods to achieve scaffolds with desired micro/nanostructure design as well as the applications of these bioinspired scaffolds. We will also discuss the current dilemma and possible development direction from a biomedical perspective.

## Graphical abstract

As an emerging field blending cell biology and material science, tissue engineering technique allows the construction of biomimetic living complexes as organ substitutes for heart repair. In this review, we will present the recent progress in cardiac tissue engineering and artificial hearts, ranging from the critical elements, advanced fabrication methods to the applications of these biohybrid engineered scaffolds.

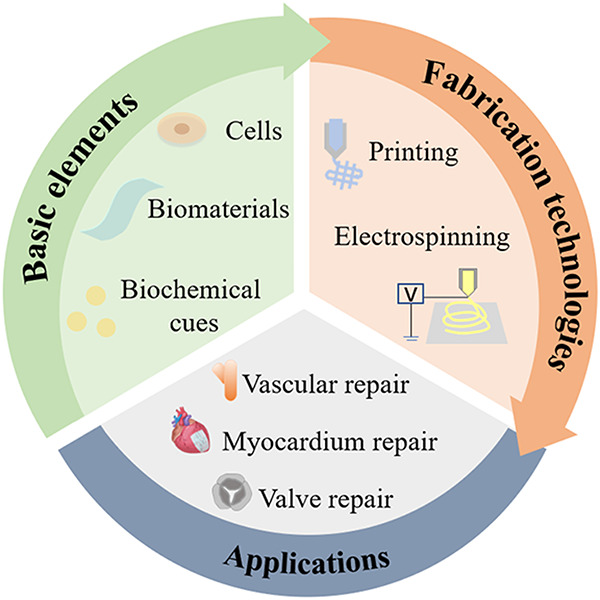

1

**Key points**

- Emerging technologies for cardiac tissue engineering are introduced.
- The applications of engineered scaffolds for heart repair are presented.
- Remaining challenges and future direction of cardiac tissue engineering are discussed.

## 2 INTRODUCTION

Heart diseases, especially cardiovascular diseases, greatly threaten the health of patients and bring heavy burden on society for their high morbidity and mortality.^1^, ^2^, ^3^, ^4^, ^5^ Organ transplantation is recognized as the only long‐term effective therapy for treating severe cardiovascular diseases.^6^, ^7^, ^8^ Nevertheless, the shortage of donors and severe immune responses greatly limit the applications of this method. In this regard, in order to maintain or even recover the physiological functions of cardiac tissues, tremendous effort has been devoted to developing alternative strategies, such as pharmacotherapy,^9^ ventricular assist devices,^10^ cell therapy,^11^, ^12^, ^13^ tissue engineering,^14^ and so on.^15^, ^16^, ^17^ Among them, cardiac tissue engineering has received great interest because of its superiority in reconstructing complex three‐dimensional (3D) spatial structures and recapitulating functions similar to in vivo ones.^18^ Up to date, cardiac tissue engineering has successfully created cardiac muscles,^19^ vascular substitutions,^20^ and heart valves,^21^ which builds a bridge between laboratory research and clinical applications.

In this review, we are intended to present the recent progress concerning cardiac tissue engineering and artificial hearts. After introducing the critical elements in cardiac tissue engineering, we enumerate advanced fabrication methods to produce the scaffolds with biomimetic micro/nanostructure design. Through typical examples, we also report the cutting‐edge applications of these biohybrid scaffolds as well as discuss their advantages and disadvantages in depth. Finally, the current dilemma and the future development direction of cardiac tissue engineering would be summarized from a biomedical perspective.

## 3 BASIC ELEMENTS OF CARDIAC TISSUE ENGINEERING

Tissue engineering is a product of interdisciplinary communication and integration, which brings new possibilities for the treatment of cardiac diseases.^14^, ^18^, ^19^, ^20^, ^21^ In this technique, several critical elements should be taken into consideration, including cell sources, biomaterial‐based scaffolds, and biochemical cues.^22^, ^23^, ^24^ Benefitting from the progress of bio‐fabrication stratagems, these elements could be effectively integrated into an entirety in a controllable manner.^25^ In the following sections, we will introduce the progress of these critical elements in cardiac tissue engineering in detail.

### 3.1 Cell sources

As an indispensable component of tissue engineering, the cell sources have received extensive investigation during the past decades.^26^, ^27^ Since the cells are intended to repair the injured site of heart, the ideal cell sources should meet the requirements of easy access, strong proliferative capacity, active functionality, weak immunological rejection, and so on. During the early stage of cardiac tissue engineering, the frequently used cell lines involve cardiomyocytes,^28^ skeletal muscle cells,^29^ fibroblasts,^30^ and vascular endothelial cells,^31^ which have shown the positive therapeutic effect on recovering the function of cardiac tissues to some extent. However, these highly specialized cells are difficult to meet the increasing demand in relevant field.

With the rise of stem cell science, the seeding cells have been greatly expanded, such as mesenchymal stem cells (MSCs),^32^ embryonic stem cells (ESCs),^33^ induced pluripotent stem cells (iPSCs),^34^ etc.^35^ These stem cells have respective advantages and limitations, which could be chosen and utilized according to practical scenes. For example, MSCs have been extensively employed in clinical applications for their features of immunity regulation, safety, and homing effect. ESCs show unparalleled proliferative capacity and totipotency, while iPSCs derived from the gene editing technique show broad prospects in personalized medicine. When applied for treating injured myocardium, stem cells could also secrete regenerative factors through the paracrine mechanism to activate the endogenous repair process, thus further enhancing the curative effect. In general, stem cells are gradually becoming popular choices for myocardial engineering, owing to their outstanding pluripotency and proliferation properties. Nevertheless, the uncontrollable differentiation direction and the risk of tumorigenicity impede the further progress of stem cells in cardiac engineering. In this regard, researchers are inclined to differentiate stem cells into target ones in vitro before transplantation.

### 3.2 Scaffolds

The scaffolds could provide physical support for the cellular component, whose features have great affections on the formation of engineered tissues. Since the scaffolds are designed for applications in vivo, they should possess excellent biocompatibility, biomimetic topography, enough mechanical strength, sound porosity, biodegradablity, and so on. Compared with the direct injection of cells, the ideal scaffolds could not only enhance the cell retention rate, but also could create an extracellular matrix (ECM)‐similar environment for promoting the adhesion, migration, differentiation, and proliferation behaviors of seeded cells. Up to date, various scaffolds composed of different materials, such as natural polymers,^36^, ^37^, ^38^, ^39^ synthetic materials,^40^, ^41^ and decellularized extracellular matrices,^42^, ^43^, ^44^ have been proposed. Among them, the natural polymers and decellularized extracellular matrices own inherent biocompatibility and biodegradability, but possess poor mechanical strength, while the synthetic materials demonstrate improved mechanical performance but have disadvantages of potential biosafety issues. Considering this, researchers tend to combine both natural and synthetic materials for fabricating scaffolds with desired properties.

**FIGURE 1:**
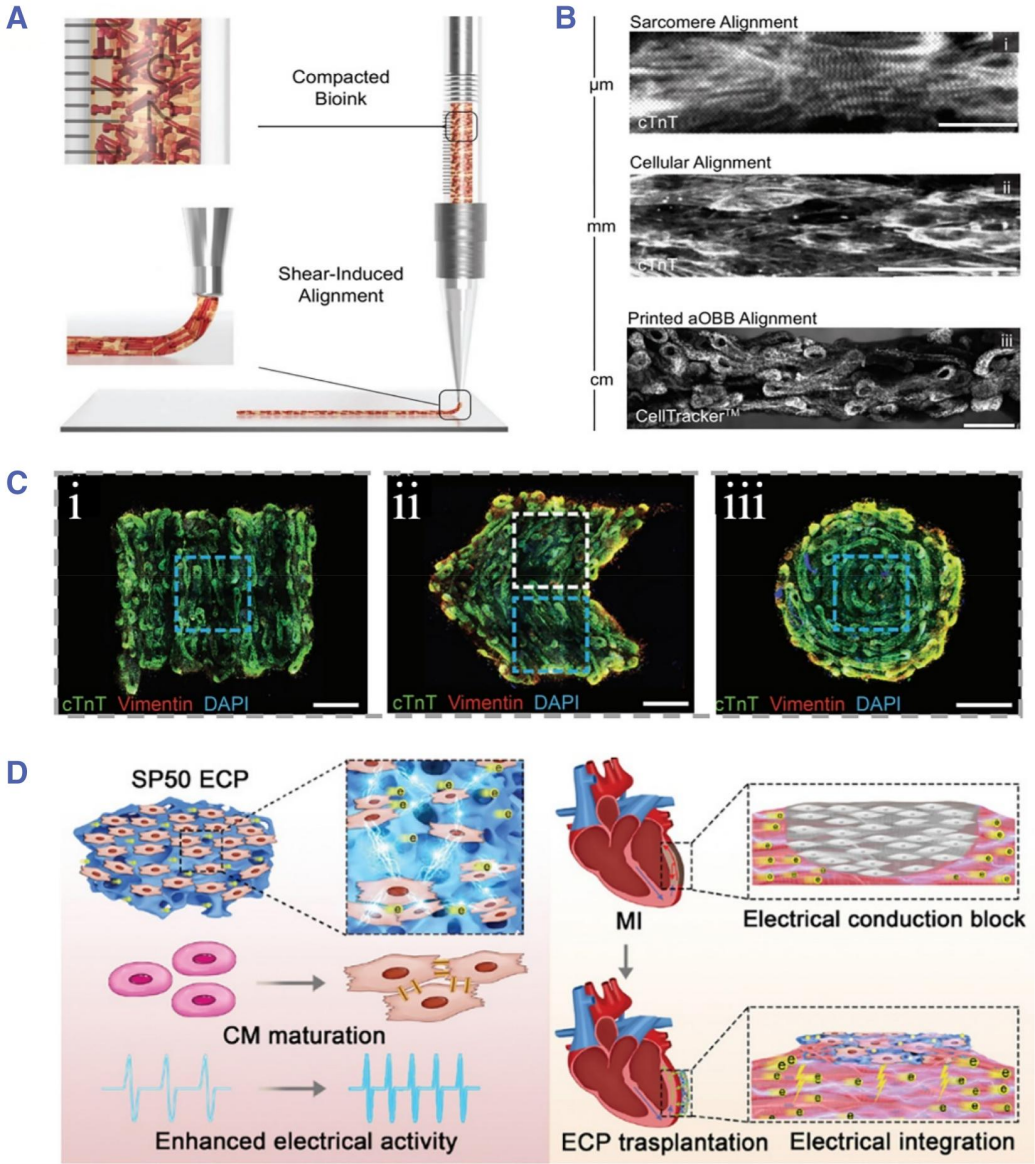
(A) The conceptual graph showing the alignment of building blocks during the bioprinting process. (B) Detailed images showing the alignment of cardiac tissues at different scales from sarcomere, cells to building blocks. (C) Bioprinting‐derived cardiac constructs composed of various featured architectures: (i) linear, (ii) chevron, and (iii) spiral ones. *Source*: (A–C) Reproduced with permission.^45^ Copyright 2022, John Wiley and Sons. (D) Schematic diagram showing the improvement of cardiomyocyte behavior under the effect of conductive scaffold. *Source*: Reproduced with permission.^46^ Copyright 2023, The Authors, published by John Wiley and Sons. MI, myocardial infarction.

Ahrens et al. described a bioprinting strategy to achieve aligned cardiac tissues by using a gelatin‐fibrinogen ECM bioink containing anisotropic organ building blocks as shown in Figure 1A.^45^ These building blocks were composed of human iPSC‐derived cardiomyocytes, which could assemble along the printing path under the action of shear and extensional forces (Figure 1B). Based on the facile bioprinting model, the cardiac constructs based on living units with different architectures, including linear, spiral, and chevron patterns, could be obtained (Figure 1C). To enhance the efficiency in treating cardiac diseases, the scaffolds are usually imparted with biomimetic topography and conductive components (e.g. gold particles, carbon nanotubes, graphene, and conductive polymers) by referring to the physiological characteristics of natural cardiac tissues. It has been reported that electrical stimulation (1–3 V) is conducive to emulate the electricity microenvironment in vivo, thus promoting the viability, beating performance, connexin expression of cardiomyocytes. Yin et al. reported novel cardiac patches by in situ polymerizing conductive polypyrrole on scaffolds of natural silk fibroin to promote the electrical activity of cardiomyocytes as shown in Figure 1D.^46^ When applied with electrical stimulation, the cell maturation situation, such as characteristic protein expression and contractile performances, was improved.

### 3.3 Biochemical cues

**FIGURE 2:**
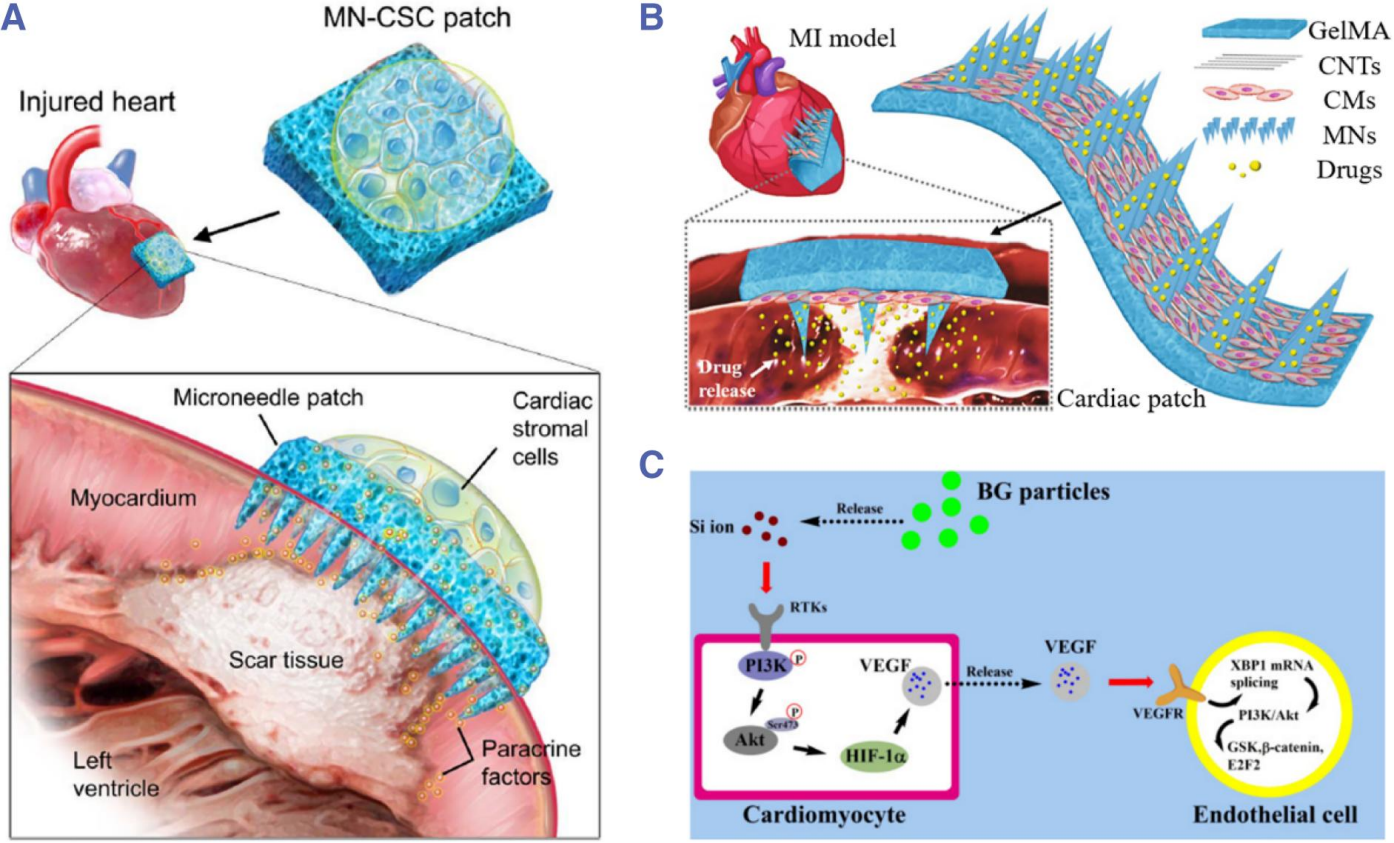
(A) The microneedle patch based on paracrine factors secreted by stem cells for cardiac treatment. *Source*: Reproduced with permission.^50^ Copyright 2018, The Authors, published by the American Association for the Advancement of Science. (B) A multifunctional cardiac patch integrated with both iPSCs‐derived cardiomyocytes and regenerative factors. *Source*: Reproduced with permission.^51^ Copyright 2021, Elsevier. (C) Schematic diagram showing the mechanism of bioactive glass on activating the angiogenesis process of cardiac tissues. *Source*: Reproduced with permission.^52^ Copyright 2021, Elsevier. MI, myocardial infarction; VEGF, vascular endothelial growth factor.

During the growth and repair process of organs, the biochemical cues, such as growth factors and other bioactive molecules, play important roles in mediating cell behaviors for functionalization. Given that, biochemical cues are usually incorporated into scaffolds to provide highly biomimetic microenvironment for cells.^47^, ^48^ To maintain the bioactivity of these molecules, the incorporating methods center on mild ones, such as covalent binding, noncovalent interactions, or simple encapsulation. In some cases, the stem cells can be regarded as a resource pool for sustained secretion of cytokines.^49^, ^50^, ^51^ Gu et al. and his team reported a cardiac patch with microneedles as channels to deliver paracrine factors from cardiac stromal cells for treatment as displayed in Figure 2A.^50^ To enhance functionality, the scaffolds could be combined with both bioactive molecules and the living cells to repair injured hearts. Our group has described a conducive microneedle patch cultured with iPSCs‐derived cardiomyocytes, which was also loaded with interleukin‐10 (IL‐10) and vascular endothelial growth factor (VEGF) for cardiac tissue engineering (Figure 2B).^51^ Note that the stem cells could be genetically engineered to express specific factors for improved therapeutic potential as demonstrated by Park et al.^53^ In addition to loading bioactive molecules into scaffolds, stimulating factors or drugs that could promote the cytokine secretion or enhance the communication of living cells have also been integrated with scaffolds.^52^, ^54^ As an example, the activation effect of bioactive glass on VEGF paracrine signaling of cardiomyocytes was investigated and proved by Shi et al., which would ultimately benefit the repair of myocardial tissue (Figure 2C).^52^ These examples illustrate the fact that the field of cardiac tissue engineering is experiencing a booming period.

## 4 FABRICATION TECHNIQUES

In recent years, attempts have been devoted to developing convenient and effective strategies for fabricating engineered cardiac scaffolds. Based on these fabrication techniques, the scaffolds with tailored component, structure, and function features could be achieved.^18^, ^19^, ^20^, ^21^ With the deepening understanding of myocardial tissues, more advanced technologies are emerging to manufacture artificial hearts that recreate the elaborate spatial details and complex micro/nanostructure of real hearts. In this section, we will focus on the cutting‐edge progress of these exciting techniques to fabricate cardiac scaffolds.

### 4.1 Electrospinning

**FIGURE 3:**
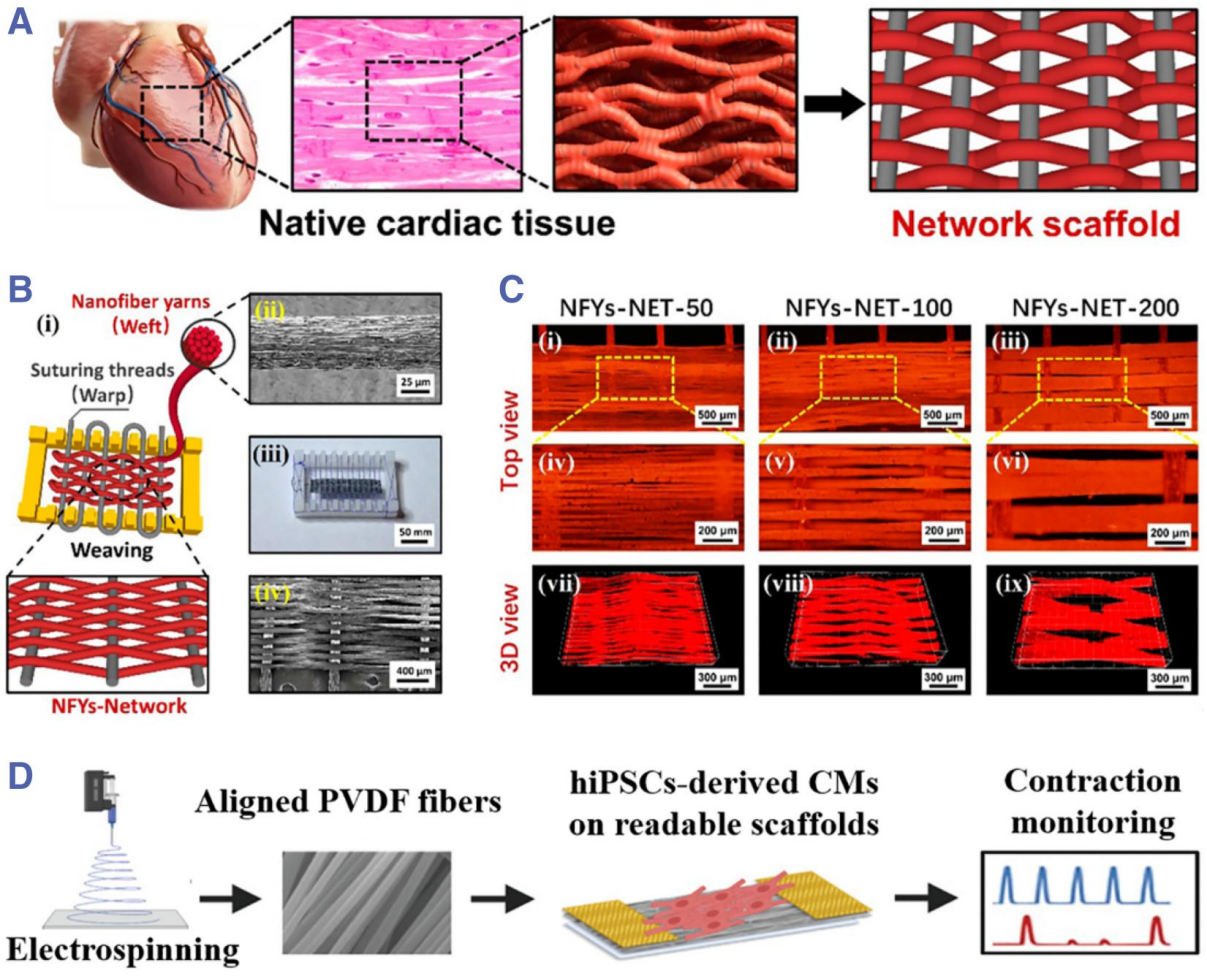
(A) The bioinspired design of anisotropic networks by referring to native cardiac tissues. (B) The weaving of electrospinning fibers based on surgical suturing threads. (C) The fibrous network composed of fibers with different diameters. *Source*: (A–C) Reproduced with permission.^56^ Copyright 2017, American Chemical Society. (D) The electrospinning PVDF scaffold for cardiomyocyte differentiation and sensing. *Source*: Reproduced with permission.^57^ Copyright 2020, John Wiley and Sons. PVDF, poly‐(vinylidene fluoride).

Electrospinning is a traditional technique to prepare microscale or nanoscale fibrous scaffolds with the virtue of mimicking the anisotropic network structure of cardiac tissues.^55^ In particular, the technique is suitable for mass production, which could meet the increasing requirement in laboratory and even in industry. To match the dimension and mechanical strength of natural tissues, the electrospinning fibers are usually woven into 3D constructs, followed by the encapsulation of hydrogel layer for cardiomyocyte cultivation. Wu et al. proposed bioinspired 3D scaffolds by weaving electrospinning nanofibers into anisotropic networks for cardiomyocyte alignment as shown in Figure 3A,B.^56^ The described nanofiber network was composed of biocompatible silk fibroin and polycaprolactone as well as conductive carbon nanotubes, whose diameters were adjustable upon changing electrospinning parameters (Figure 3C). After encapsulating with a hydrogel shell, such scaffolds were further demonstrated with positive influences on cardiomyocyte orientation and maturation.

More importantly, the cardiac scaffolds could not only serve as repairing material, but also appropriate candidates for assessing the functionality of engineered cardiac tissues before and after transplantation, through embedding sensors or directly employing stimuli‐responsive raw materials for scaffold construction.^58^ For this purpose, Adadi et al. proposed an electrospinning scaffold composed of piezoelectric poly‐(vinylidene fluoride) (PVDF) material as schemed in Figure 3D.^57^ Such scaffold with aligned PVDF fibers was utilized for the differentiation of iPSCs into cardiomyocytes and induced their alignment. In particular, the PVDF‐based system could convert the mechanical activities of cardiomyocytes into regular voltage transients, thus providing a facile strategy for real‐time biosensing. In addition to engineered myocardium, studies in this area are also focused on reappearing other critical constitutions of heart, such as vascular structures^20^ and cardiac valves.^21^, ^59^ As an example, Duan and his team reported an engineered nanofibrous heart valve with comparable mechanical features to native ones, relying on the positive/negative conjugate electrospinning technique.^59^ They found that the introduction of poly(aspartic acid) was beneficial to improving the functions of valvular interstitial cells, such as adhesion, proliferation, etc., which may provide guidance for the design of novel cardiac scaffolds.

### 4.2 Focused rotary jet spinning

**FIGURE 4:**
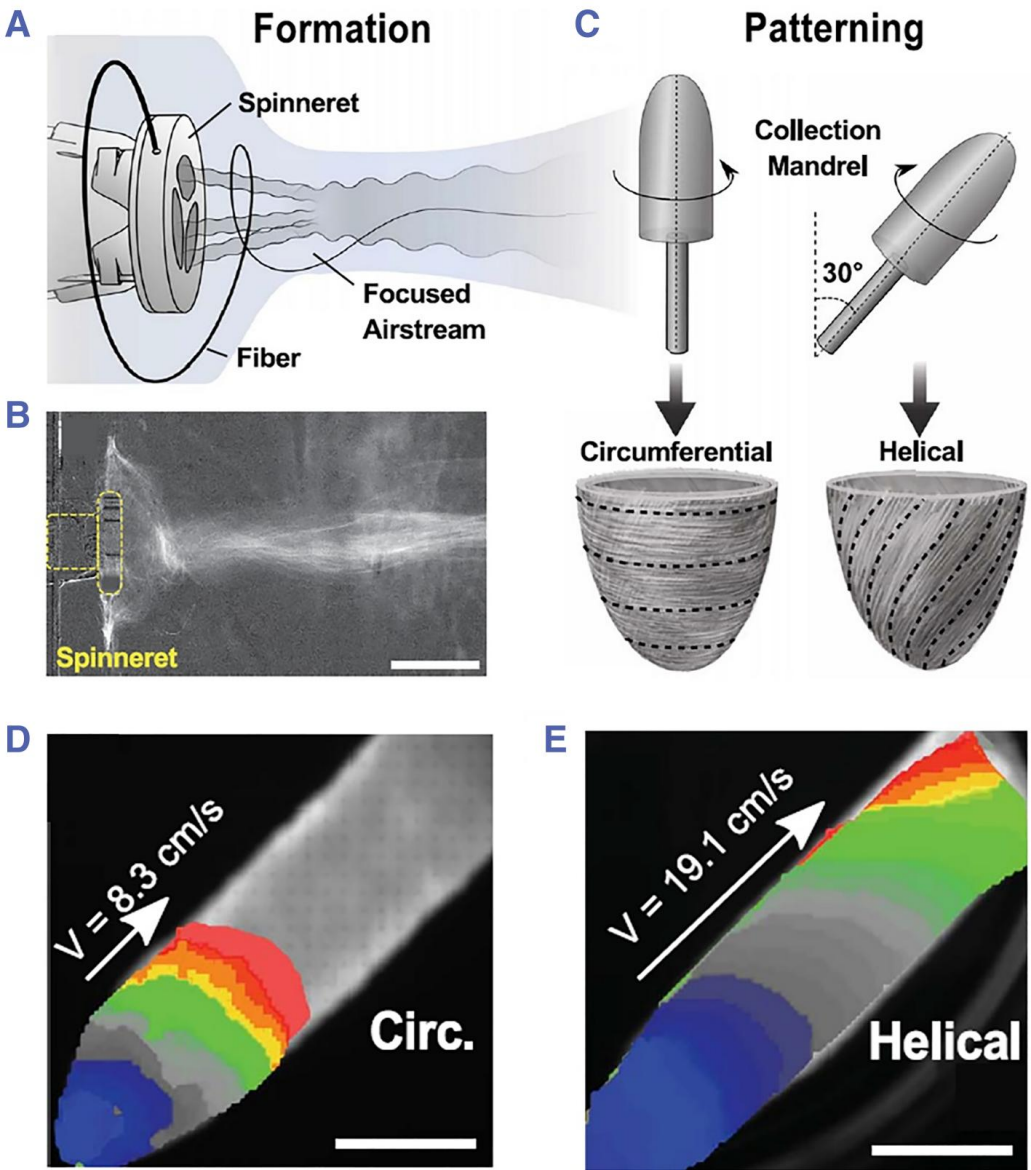
The schematic diagram (A) and differential contrast projection image (B), showing the formation phase of separated single fiber from focused airstream. (C) The patterning of fibers into circumferential or helical morphologies. (D and E) The calcium propagation of cardiomyocytes on scaffolds with circumferential fiber alignment (D) or helical fiber alignment (E). *Source*: (A–E) Reproduced with permission.^60^ Copyright 2022, The Authors, published by the American Association for the Advancement of Science.

Although various methods have been developed and considerable achievements in cardiac scaffolds have been achieved, it remains challenging to fabricate biomimetic micro/nanostructures with a high resolution within a short period. Recently, Chang et al. and his coworkers developed a novel‐focused rotary jet spinning for implementing the bioinspired helical structure into cardiac scaffolds with the aim of investigating the role of helical alignment in heart functions as shown in Figure 4A–C.^60^ This technique could generate the polymeric fibers based on centrifugal jet spinning, which were then deposited on a convex‐structured mandrel with desired geometries by manipulating the airstream. After seeded with primary or induced cardiomyocytes, the scaffolds could help the anisotropic alignment and subsequent spontaneous contractions of cells. Further calcium propagation results demonstrated that helical alignment had faster conduction velocities than circumferential alignment (Figure 4D,E), thus revealing the importance of alignment in excitation‐contraction coupling. In general, novel approaches for producing engineered cardiac scaffolds in a more controllable, more elaborate, and rapider manner are constantly springing up. It could be anticipated that these biohybrid scaffolds could bridge the gap between laboratory research and clinical applications in the future.

### 4.3 3D printing

**FIGURE 5:**
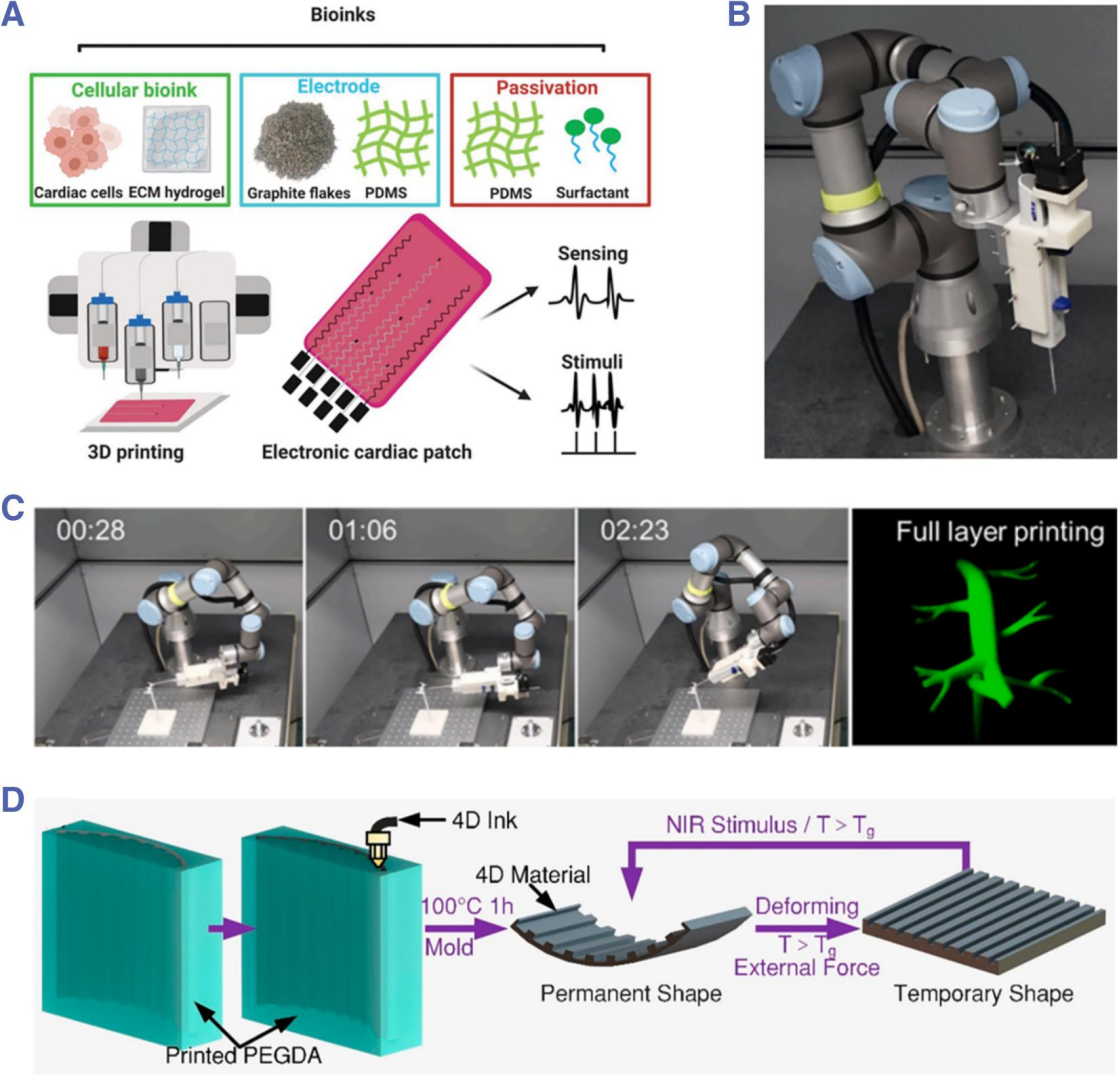
(A) The fabrication scheme of the cardiac patch with built‐in electronics. *Source*: Reproduced under terms of the CC‐BY license.^66^ Copyright 2021, The Authors, published by John Wiley and Sons. (B) Optical micrograph of the robotic arm designed with six flexible joints. (C) The robotic printing system for engineering cardiac tissues onto vascular scaffolds. *Source*: (B, C) Reproduced with permission.^67^ Copyright 2022, Elsevier. (D) The fabrication and programming procedures of the 4D printed scaffold with aligned patterns. *Source*: Reproduced with permission.^68^ Copyright 2021, American Chemical Society. NIR, near‐infrared.

Since its emergence, 3D printing has been regarded as a promising strategy to produce functional materials with complicated component and morphology design.^61^ In this technique, the living cells and biomaterial pregels could constitute the bioink for one‐step construction of engineered scaffolds. Considering the intrinsic electrical conduction feature of native cardiac tissues, conductive cues, such as titanium carbide (MXene),^62^ graphene‐based materials,^63^ gold nanoparticles,^64^ and so on,^65^ are usually incorporated into the scaffold for facilitating or even monitoring the function maturity of engineered myocardium. By integrating with a planar electronic system, Asulin et al. created a novel cardiac patch from additive manufacturing as displayed in Figure 5A.^66^ Because of the built‐in electronics, the engineered tissue could be stimulated under control, whose reactions could also be detected by the same system. To enhance the functionality of fabricated scaffolds, much effort has been devoted to developing novel ones with vascularized structures. Zhang et al. designed a robotic arm consisting of six flexible joints for 3D printing of living cardiac tissues on vascular scaffolds (Figure 5B,C).^67^ During the process, the favorable cellular adhesion was mediated by hydrophobic force based on oil environment. Note that the authors also developed a bioreactor perfusion system for nutrient supply, in which case the viability of cells could maintain over 6 months. In recent years, the concept of 4D printing has attracted great interest in the field of tissue engineering. Wang et al. proposed 4D cardiac patch‐aligned patterns for cell alignment and programmable curvature by employing the digital light processing strategy (Figure 5D).^68^ Upon near‐infrared (NIR) irradiation, the soft scaffolds would generate curvature variations, thus realizing the uniform distribution of cardiomyocytes and expanding their practical values in cardiac repair.

## 5 APPLICATIONS OF ENGINEERED SCAFFOLDS

### 5.1 Myocardial infarction (MI) treatment

**FIGURE 6:**
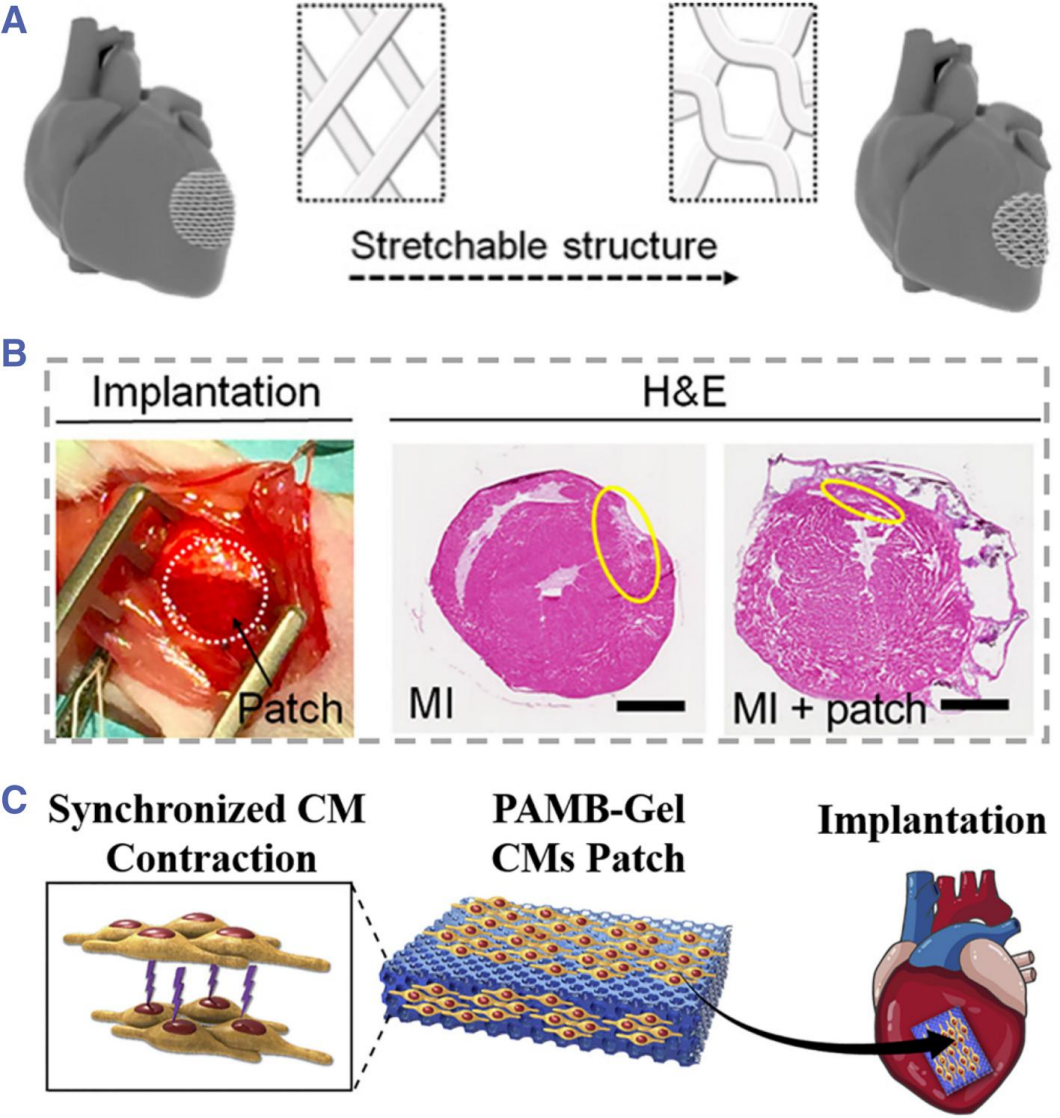
(A) Schematic diagram showing the stretchable structure of the physiologically adaptable patch. (B) The implantation and therapeutic effect of the patch in the MI model. *Source*: (A and B) Reproduced with permission.^73^ Copyright 2020, The Authors, published by the American Association for the Advancement of Science. (C) Conceptual graph showing the implantation of conductive patch for MI treatment. *Source*: Reproduced with permission.^74^ Copyright 2020, Elsevier. MI, myocardial infarction.

Benefitting from the development of biological science and material science, these engineered scaffolds loaded with cells and biochemical cues have found broad prospects in recovering the morphology and function of cardiac tissues.^19^, ^20^, ^21^ Among various heart‐related diseases, myocardial infarction (MI) would cause irreversible myocardial necrosis of patients, which has been regarded as a troublesome clinical issue.^69^, ^70^ Given this insight, various engineered scaffolds with different forms, such as particles^71^ and patches,^50^, ^51^, ^72^ have emerged for repairing the injured myocardium. When developing the scaffolds of myocardium repair, there are several aspects that should be taken into consideration, including excellent electrical conductivity, effective drug incorporation, appropriate mechanical strength, biomimetic surface morphology, etc. Considering these principles, Cui et al. proposed a 4D physiologically adaptable patch with enhanced mechanical tolerance that could generate a reversible switch between wavy status and mesh status during cardiac beating cycles (Figure 6A).^73^ By applying an incorporated stimulation of mechanical stain and shear force, the cardiomyocytes cultured on the patch demonstrated enhanced vascularization maturation. After implantation for chronic MI model treatment, the described biohybrid patch could avoid the remodeling of left ventricle (Figure 6B). Compared with traditional 3D constructs, the 4D scaffolds exhibit superior adaptability and functionality, which provide new possibility for the development of tissue engineering. To improve the electrical integration of engineered scaffolds with myocardium, Chen et al. developed a conductive cardiac patch seeded with cardiomyocytes as schemed in Figure 6C.^74^ The conductive polymer component of patch could promote the propagation of electrical pulse in the injured region, while the cardiomyocytes on scaffolds could replace the lost ones to recover the cardiac function.

### 5.2 Vascular repair

**FIGURE 7:**
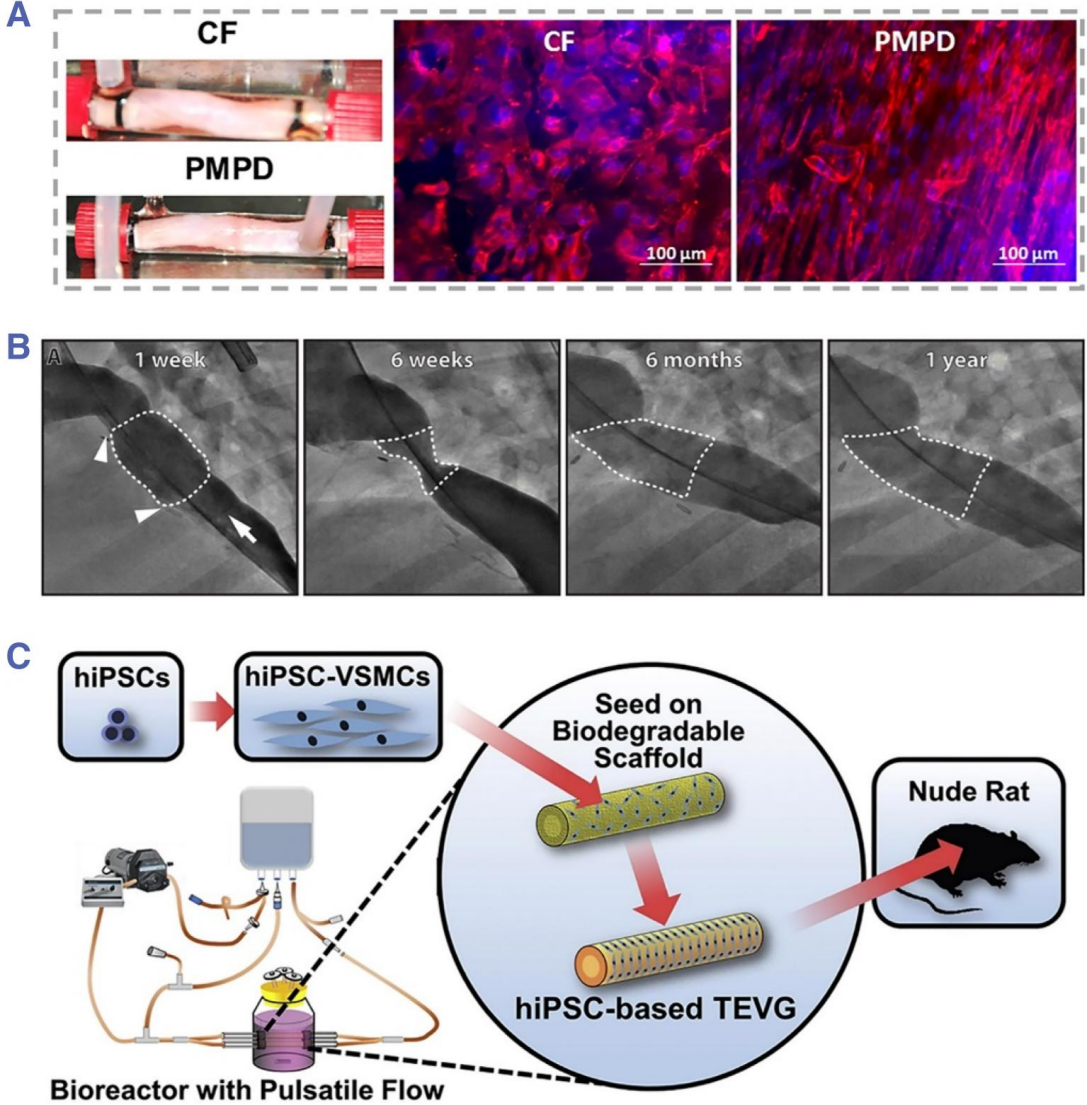
(A) The optical images of the grafts from bioreactors with different parameters (left) and the fluorescent images showing the morphology of endothelial cells on the grafts with CF and PMPD stimulations (right). *Source*: Reproduced with permission.^83^ Copyright 2021, John Wiley and Sons. (B) Angiographic images showing the narrowing and recovery of vascular graft in an ovine model. *Source*: Reproduced with permission.^84^ Copyright 2020, The Authors, published by the American Association for the Advancement of Science. (C) Schematic diagram of the human iPSCs‐based scaffold for vascular repair. *Source*: Reproduced with permission.^85^ Copyright 2020, Elsevier. CF, constant frequency; PMPD, physiologically modeled pulse dynamics; TEVG, tissue‐engineered vascular graft.

Nowadays, cardiovascular diseases have become a serious health threat for human health globally. The high incidence of cardiovascular diseases could be mainly attributed to atherosclerosis that caused by various factors, including life pressure, unhealthy habits, environmental pollution, etc.^75^, ^76^, ^77^ Clinically, intervention therapies, such as percutaneous coronary intervention, are usually utilized for treating the vascular narrowing.^78^, ^79^ However, such therapies are faced with the problem of limited transplantation sources. In this regard, vascular grafts based on engineered biomaterials have been extensively investigated and developed to replace the autografts.^80^, ^81^, ^82^ Based on decellularized human umbilical veins, Van de Walle et al. developed a novel strategy to facilitate the recellularization and remodeling of vascular grafts through flow stimulation.^83^ Compared with the constant frequency, variable flow frequencies (physiologically modeled pulse dynamics) facilitated the confluence and alignment of cells on the graft as shown in Figure 7A. This instance reveals the importance of mechanical stimulation similar to physiological conditions on the function maturation of engineered scaffolds.

Apart from decellularized scaffolds, the engineered vascular grafts composed of synthetic materials are emerging as the mainstream. In a work by Drews et al., they reported a degradable vascular graft of poly(glycolic acid) (PGA) and caprolactone/lactide (PCLA), which were seeded with mononuclear cells from bone marrow for the investigation of early stenosis mechanisms.^84^ They found that computational model could greatly predict the early stenosis phenomenon as happened in clinical experiments. Interestingly, the computational model also indicated that this graft narrowing could reverse under the mediation of inflammation, which was validated by an ovine model (Figure 7B). Such synthetic component could endow the scaffold with adjustable mechanical strength and degradation rate, while the computational model could provide guidance for the design of scaffolds. Utilizing human iPSCs as cell sources, Luo et al. also proposed a kind of vascular grafts made of biodegradable polymers with comparable mechanical performance to in vivo vessels (Figure 7C).^85^ After cultivation of human iPSCs‐derived vascular smooth muscle cells, the mechanical intensity of grafts was enhanced through stretching‐induced collagen deposition and proliferation of seeding cells with the aim of better implantation effect.

### 5.3 Valve regeneration

**FIGURE 8:**
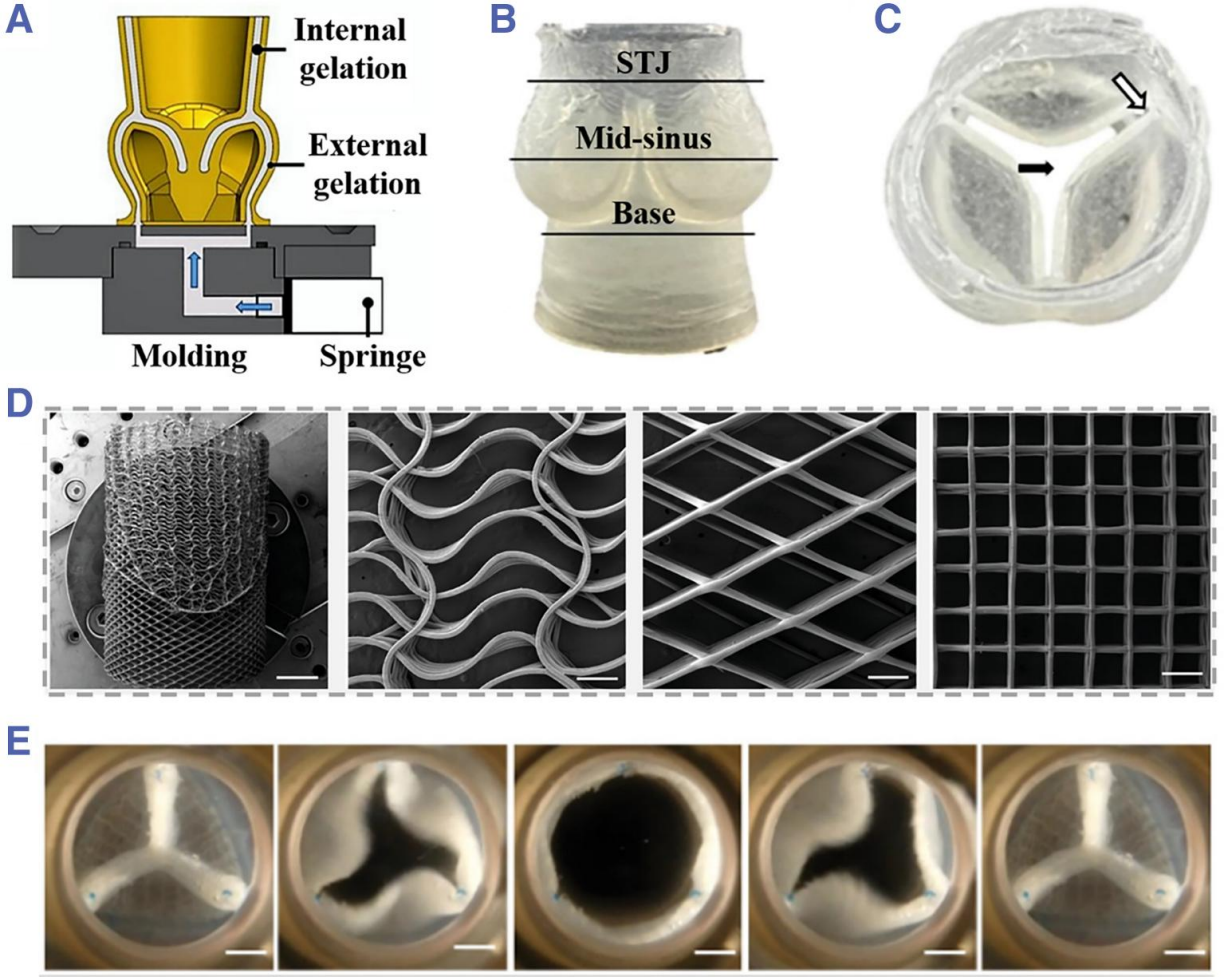
(A) The schematic diagram showing the fabrication of hydrogel valve from a sugar glass mold. (B and C) The side view (B) and top view (C) of the aortic valve scaffold composed of sodium alginate (STJ, sino tubular junction). *Source*: (A–C) Reproduced under terms of the CC‐BY license.^89^ Copyright 2022, The Authors, published by MDPI. (D) SEM images showing the tubular scaffold with spatially heterogeneous patterns. (E) Systolic hemodynamic performance testing of the heterogeneous tubular scaffold. *Source*: (D and E) Reproduced with permission.^90^ Copyright 2022, The Authors, published by John Wiley and Sons.

As another cause of cardiovascular disease mortality, heart valve diseases have achieved great attention in recent years.^86^ Up to date, valve replacement is the only effective method for improving the cardiac functions of patients with valve diseases.^87^ Since the traditional mechanical substitute would cause complications, including thromboembolism, hemolysis, and so on,^88^ tissue‐engineered valves are emerging as alternative grafts for valve repair or serving as in vitro models for studying the underlying mechanisms of valve diseases.^21^, ^89^, ^90^ Rioux et al. developed a biofabrication technique for stable production of 3D scaffolds reappearing the native geometry of aortic valves.^89^ In their work, the sodium alginate hydrogel was employed as the main component of engineered scaffold, which was replicated from a dissolvable mold with customed morphology as shown in Figure 8A–C. When applied for cardiac bioreactor testing, the flow rate and pressure distribution inside these valve grafts demonstrated to be similar with physiological situation with little individual difference. To achieve engineered valves with improved cellular infiltration, Saidy et al. demonstrated the application of melt electro‐writing technique for fabricating tubular scaffolds, which were imparted with spatially heterogeneous patterns inspired by leaflets and interleaflet triangles (Figure 8D).^90^ After integration into the elastin‐like matrix, the derived valve substitute displayed remarkable systolic hemodynamic functions for aortic condition (Figure 8E). In addition to the improvement of structural features, attempts in tissue‐engineered valves are also focused on adopting natural biomaterials existing in native valves. For instance, Lei et al. incorporated hyaluronic acid, which is naturally contained in the valve ECM, into fibrin‐based scaffolds to reduce retraction induced by interstitial cells.^88^

## 6 PERSPECTIVE AND CONCLUSIONS

In summary, we have overviewed the recent progress of cardiac tissue engineering including the basic components in tissue engineering, the emerging fabrication techniques for tissue‐engineered scaffolds, and their applications. With the efforts of multidiscipline experts, the engineered scaffolds have been imparted with ECM‐like constituents and bioinspired delicate nano/microstructures. Compared with traditional methods, the cardiac tissue engineering technique has the advantages of forming living substitutes with multifunctionality and comparable mechanical property to native tissues, thus becoming ideal candidates for repairing injured tissues of heart.

Although with great achievements, cardiac tissue engineering is still faced with challenges for further commercial and clinical transformation. Firstly, the viability maintenance and electromechanical integration of seeding cells with host cells after implantation remain to be explored. In this regard, organs‐on‐chips could be utilized as ideal research platforms to investigate the factors or mechanisms determining these processes,^91^, ^92^ thus proving guidance for safe and effective transplantation of engineered tissues.

The second issue is about improving the component and structure complexity of scaffolds. During the optimization of scaffolds, a critical challenge is the vascularization and perfusion efficiency when they are applied for myocardium treatment. For this purpose, attempts have been devoted to embedding vascular channels into the scaffold^93^ or adding angiogenic factors to promote the spontaneous vascular formation of cardiac tissues.^51^, ^52^ In future, generating dense vascular network regenerated for cardiac tissues may depend on the technological advances of these methods, such as high‐resolution 3D printing and organoid techniques, together with the appropriate combination of them.

The third issue refers to the mass production and regulated production of engineered scaffolds. So far, the fabrication of cardiac scaffolds is still in the laboratory stage, lacking of standardized and uniform production protocols. This may lead to the uncontrollable quality and limited therapeutic effect of engineered scaffolds during the application, whose transformation toward commercial products should rely on the cooperation of academic institutions, pharmaceutical companies, and government impartments. Although challenges remain, we anticipate that our overview could give inspiration to the research fellow in related subjects, thus driving the advance of cardiac tissue engineering technique.

## AUTHOR CONTRIBUTIONS

Yuanjin Zhao conceived the idea; Lingyu Sun wrote the manuscript; Yu Wang and Dongyu Xu revised the manuscript.

## CONFLICT OF INTEREST STATEMENT

The authors declare no competing financial interests. Yuanjin Zhao is a member of the *Smart Medicine* editorial board.
